# Identifying molecular targets for reverse aging using integrated network analysis of transcriptomic and epigenomic changes during aging

**DOI:** 10.1038/s41598-021-91811-1

**Published:** 2021-06-10

**Authors:** Hwang-Yeol Lee, Yeonsu Jeon, Yeon Kyung Kim, Jae Young Jang, Yun Sung Cho, Jong Bhak, Kwang-Hyun Cho

**Affiliations:** 1 Department of Bio and Brain Engineering, Korea Advanced Institute of Science and Technology (KAIST), Daejeon, 34141 Republic of Korea; 2 Genome Research Institute, Clinomics Inc, Ulsan, 44919 Republic of Korea; 3 Department of Biomedical Engineering, College of Information and Biotechnology, Ulsan National Institute of Science and Technology (UNIST), Ulsan, 44919 Republic of Korea; 4 Korea Genomics Center (KOGIC), Ulsan National Institute of Science and Technology (UNIST), Ulsan, 44919 Republic of Korea; 5 Personal Genomics Institute (PGI), Genome Research Foundation (GRF), Osong, 28160 Republic of Korea

**Keywords:** Gene regulatory networks, Regulatory networks, Systems analysis, Ageing

## Abstract

Aging is associated with widespread physiological changes, including skeletal muscle weakening, neuron system degeneration, hair loss, and skin wrinkling. Previous studies have identified numerous molecular biomarkers involved in these changes, but their regulatory mechanisms and functional repercussions remain elusive. In this study, we conducted next-generation sequencing of DNA methylation and RNA sequencing of blood samples from 51 healthy adults between 20 and 74 years of age and identified aging-related epigenetic and transcriptomic biomarkers. We also identified candidate molecular targets that can reversely regulate the transcriptomic biomarkers of aging by reconstructing a gene regulatory network model and performing signal flow analysis. For validation, we screened public experimental data including gene expression profiles in response to thousands of chemical perturbagens. Despite insufficient data on the binding targets of perturbagens and their modes of action, curcumin, which reversely regulated the biomarkers in the experimental dataset, was found to bind and inhibit JUN, which was identified as a candidate target via signal flow analysis. Collectively, our results demonstrate the utility of a network model for integrative analysis of omics data, which can help elucidate inter-omics regulatory mechanisms and develop therapeutic strategies against aging.

## Introduction

Aging is a process that accompanies external (physical appearance) and internal (physiological functions) changes in an organism, which are affected by molecular interactions across multiple omics layers. Clinical studies of human aging have identified aging-related biomarkers associated with metabolic disorder^1^, loss of skeletal muscle^2^, neurodegeneration^3^, skin wrinkles^4^, and hair loss^5^ as well as an increased risk of aging-related diseases^6,7^ such as type 2 diabetes, cancers, and Alzheimer’s disease. With the development of next-generation sequencing (NGS) technologies, molecular genetic studies have accumulated large quantities of omics data on aging and aging-related diseases^7–9^.

In particular, blood is easier to collect from living human body than other tissues, and also actively studied in forensic science to estimate the age of suspects^10,11^. Based on the advantages, numerous association studies on human aging have conducted with blood samples, and revealed aging-related changes in DNA methylation^12–16^ and gene expression^15–19^, and their application as biological clocks to estimate chronological age from the identified aging signatures^20,21^. However, the causal relationship between variations in biomarkers and the specific cellular dysfunction or phenotypes related to aging remains largely unknown. To investigate these molecular mechanisms and causal relationships, molecular dynamics studies have been conducted to predict and control gene expression or protein phosphorylation changes in diseases^22–26^ such as cancer and diabetes from the perspective of network biology. Several studies have employed ordinary differential equations^22,23^ or Boolean logical models^24–26^, which require specific parameter fitting or logical rule inference using accumulated experimental data. Recently, a signal flow analysis^27,28^ method was developed, in which the influence of signals of perturbed nodes on the activity changes of the other nodes can be estimated based on the network structure and mode of action of each edge.

For integrative analysis, investigating the changes in methylation and gene expression in a network model requires understanding the causal relationships between two different omics layers. Changes in DNA methylation states and mRNA expression levels are closely related^29^. Typically, CpG methylations occurring in TFBSs regulate target gene expression by inhibiting or promoting the binding of specific transcription factors (TFs)^30^. Furthermore, a decrease in the expression of a gene involved in methylation/demethylation can cause changes in the overall methylation pattern^31^. It is difficult to determine which methylation/expression event is the leading cause and which is the result of the causal relationship represented in the feedback structures, as DNA methylation and gene expression are regulated by each other.

In this study, we assumed that the gene regulatory network is a closed system and that DNA methylation events are external input signals. Based on this assumption, we investigated the regulation of differentially expressed gene (DEG) markers, which results from changes in the methylation states of differentially methylated position (DMP) markers. First, we conducted paired methyl-seq and RNA-seq analysis of blood samples obtained from 51 Korean individuals aged 21–74 years and identified the DMP and DEG markers associated with age-related changes in methylation states or expression levels. Based on the blood gene regulatory network (GRN) constructed using a tissue-specific network inference study^32^, we reconstructed an aging-related gene regulatory subnetwork that can explain the changes in gene expression identified via RNA-seq. We further performed signal flow analysis to explore the mechanism by which the methylation states of DMP markers regulate the expression of DEG markers. Furthermore, via signal flow analysis, we identified candidate molecular targets that can reversely regulate the aging-related gene expression changes.

## Results

### Identification of aging-related DMPs and DEGs

**Figure 1:**
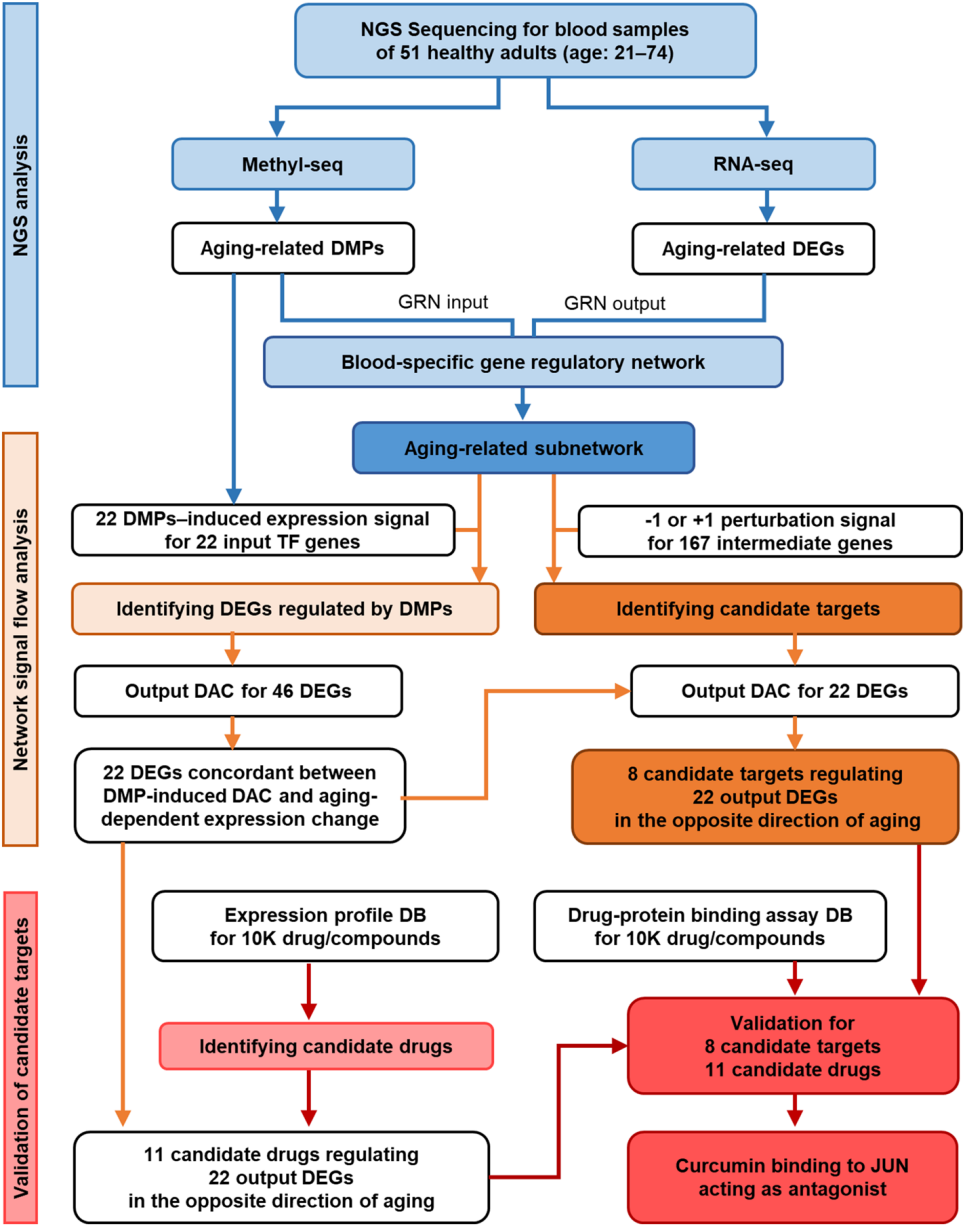
Schematic workflow of multi-dimensional investigation for identifying candidate targets to reversely regulate the DEG markers.

**Figure 2:**
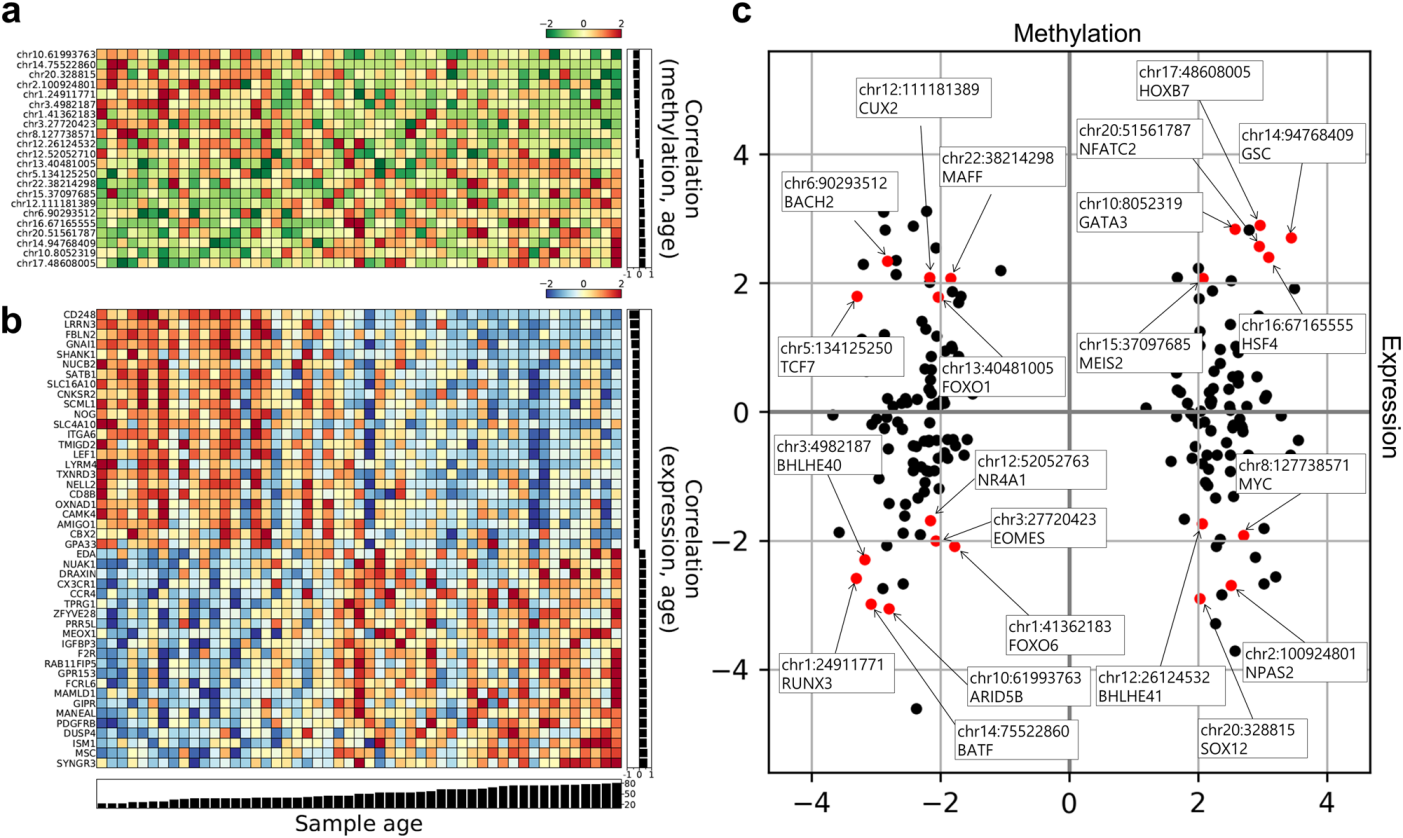
Aging-related DMP and DEG markers identified via NGS. Blood samples were collected from 51 individuals for NGS. (**a**, **b**) Heatmap of 22 DMP markers and 46 DEG markers in the aging-related gene regulatory network (GRN). (**c**) Correlation between the methylation states of DMP markers and the expression levels of neighboring genes. The 22 input DMP markers in the GRN model are indicated as red circles. DEG, differentially expressed gene; DMP, differentially methylated position.

A schematic workflow of multi-dimensional investigation in this study is shown in Fig. 1. NGS analysis was conducted using blood samples obtained from 51 healthy Korean individuals aged 20–74 years. Methyl-seq analysis identified 2,682,537 methylated CpGs from each sample. Of these, the beta values of 222,032 DMPs significantly (P < 0.05) increased or decreased according to age. These DMPs included all 32 aging-related epigenetic markers identified from 100 Korean and 300 Polish samples^33^ (hypergeometric test, P = 2.3e−35). To use the DMP markers whose methylation states are correlated with expression levels of TF genes as inputs in signal flow analysis of the GRN, we calculated the correlation between the methylation states of each DMP marker and the expression levels of nearby TF genes. The expression levels of 29 TF genes were found to be significantly correlated with the methylation states of 127 DMP markers (P < 0.05). When multiple DMPs were correlated with the same gene, the DMP with the most significant correlation was selected as a representative for calculating the expression level of the gene based on the methylation state of the DMP (Fig. 2c, Supplementary Table S1).

In RNA-seq analysis, we measured the expression levels of 19,229 protein-coding genes in each sample, and found 188 significantly up- or down-regulated DEGs according to age (adjusted P < 0.05; Supplementary Table S2). Of these, 20 DEGs overlapped with 50 aging-related transcriptomic markers identified through a meta-analysis of the blood transcriptome^19^ (hypergeometric test, P = 8.3e−28).

### Reconstruction of an aging-related GRN

**Figure 3:**
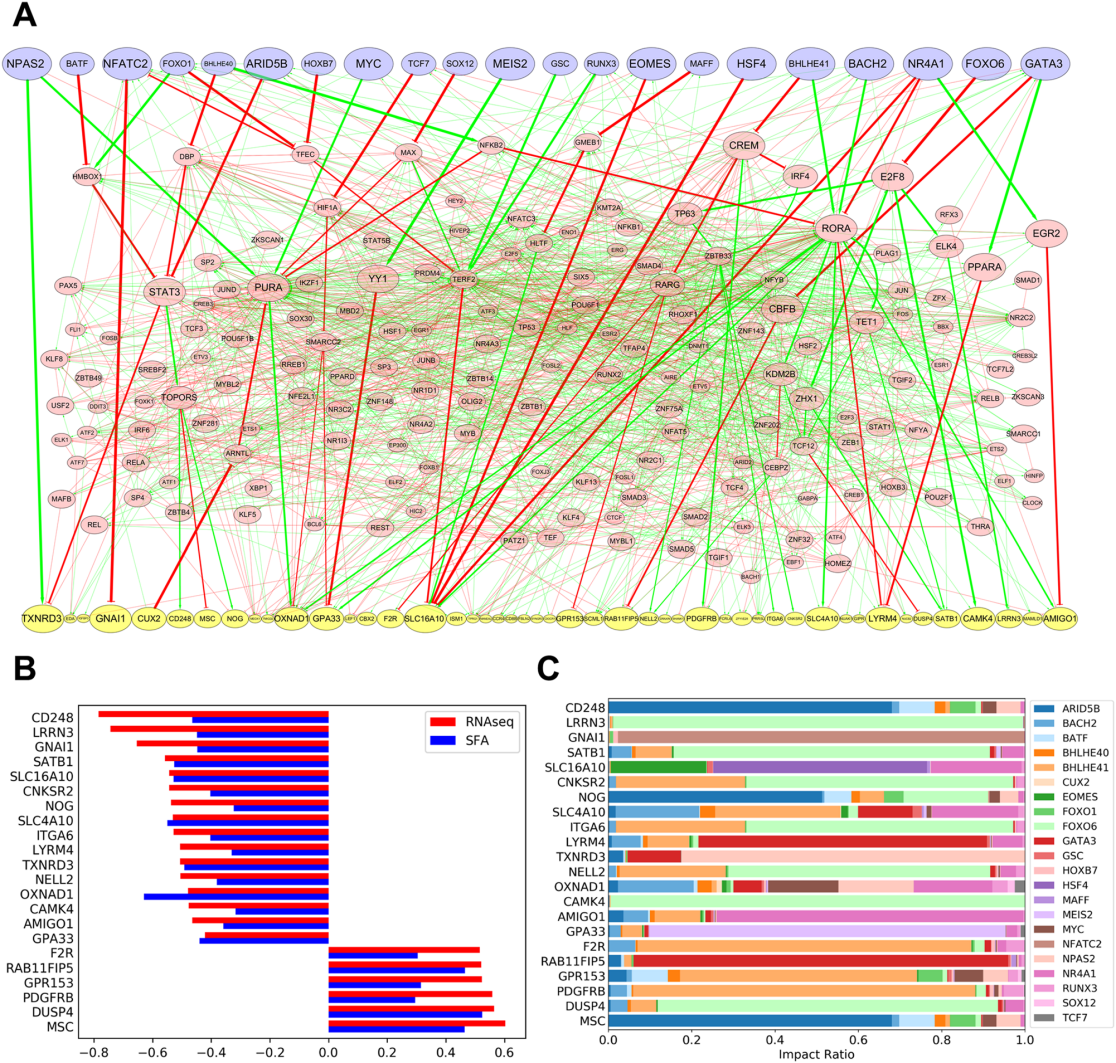
Signal flow analysis of the aging-related GRN constructed with 22 DMP markers and 46 DEG markers. (**A**) Methylation changes during aging were considered as initial signals for visualization of the aging-related GRN. An increase in edge thickness and node size is based on the intensity of the signal transmitted to the DEG markers via the network. DMP input nodes are indicated in blue; DEG output nodes are indicated in yellow; edges representing up-regulated and down-regulated TG expression are indicated in green and red, respectively. (**B**) The direction of activity change (DAC) and aging-related fold change in RNA-seq analysis were consistent for the 22 output DEGs. (**C**) Relative influence of the 22 input DMPs on the DAC of the 22 output DEGs. SFA, signal flow analysis.

To investigate the mechanism underlying how DMP-derived TF expression regulates downstream DEGs through the network, we constructed an aging-related, blood tissue-specific, GRN. Based on a previously constructed blood GRN—comprising 398,283 regulation events between 15,293 genes—in a tissue-specific network inference study^32^, we reconstructed a subnetwork composed of DMP, DEG markers, and the intermediate nodes via which DMPs regulate downstream DEGs. After filtered out genes and regulations whose expression change or regulatory relationship is not significant based on our RNA-seq experiment, the final aging-related gene regulatory subnetwork was composed of 1,198 regulations among 235 genes (Fig. 3A), including 22 input DMPs (Fig. 2a) and 46 output DEGs (Fig. 2b).

### Effect of differentially methylated TFs on differentially expressed target genes

To investigate the regulation of the transcriptome layer induced by changes in the epigenomic layer, we performed signal flow analysis, which can predict the direction of activity changes (DAC) of network nodes affected by a given initial signal input. The age-dependent changes in methylation of DMP markers were used as signal input, and their effect on DEG markers via the reconstructed GRN were predicted as DACs. As GRN concerns regulation of gene expression between TFs and their target genes, the initial signal of DMP markers were converted into expression levels of relevant TF genes based on the correlation between DMP marker methylation states and neighboring TF expression levels (Fig. 2c). As a result of signal flow analysis performed on 51 blood samples, the predicted DACs of 26 out of 46 DEGs were significantly altered according to age (P < 0.05). Of these 26 DEGs, the DACs of 22 DEGs were consistent with the increasing/decreasing directions of the DEG markers identified via RNA-seq (Fig. 3B, Supplementary Fig. S1). The remaining four DEGs were inconsistent with the RNA-seq results, although the DAC increased or decreased in a certain direction according to age (Supplementary Figs. S2, S3A). The remaining 20 DEG markers were not compared with the RNA-seq results because their DACs showed nonsignificant correlation with age (P > 0.05; Supplementary Fig. S3B). The 22 DEG markers whose DACs by methylation input were significant and consistent with the RNA-seq results were considered adequately regulated via signal flow analysis of the constructed network model. We accordingly used these markers as targets for reverse regulation to delay or attenuate aging. The relative influence of each DMP in determining the DAC of 22 DEGs was further compared (Fig. 3C).

### Candidate molecular targets and drugs to reverse aging-related DEG expression

**Figure 4:**
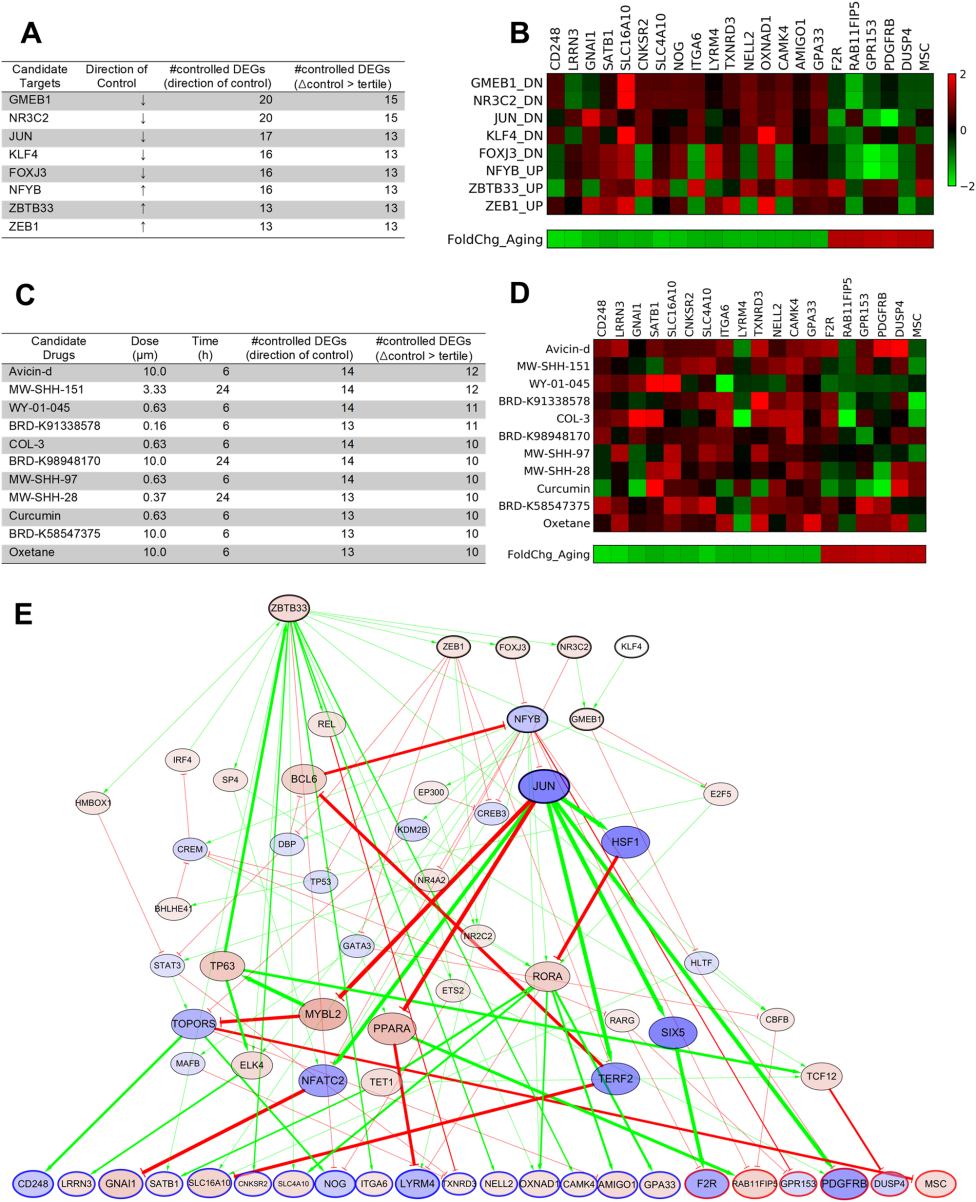
Candidate target genes identified by signal flow analysis and candidate drugs screened from the LINCS database. (**A**) List of candidate molecular targets predicted to reversely regulate the aging-related DEG expression. (**B**) Heatmap of the calculated DACs of the output DEGs for the candidate molecular target. (**C**) List of candidate drugs identified from the LINCS database that reversely regulate the DEG markers. (**D**) Heatmap of the observed expression changes of the output DEGs induced by candidate drugs. (**E**) Subnetwork visualization of candidate targets that reversely regulate the aging-related DEG expression, considering the initial signal that down-regulates JUN (blue). Signals from the candidate target genes (nodes with a black bold boundary) are transmitted via the network with up-regulating (red) or down-regulating (blue) downstream genes, and eventually regulate the aging-related expression of the output DEGs (the color of boundary denotes the expression fold change during aging).

We investigated candidate molecular targets that reversely regulate the aging-related DEG expression. We performed signal flow analysis assuming up-/down-regulation of each intermediate node as an input signal, and ranked the candidate targets based on the number of DEGs whose DAC are calculated in the opposite direction to its aging-related expression change. We identified top 5% of them as candidate molecular targets (Fig. 4A). Downregulation of GMEB1 or NR3C2 were predicted to reversely regulate 20 of 22 output DEGs, and 15 of them more significantly compared to other input signals. We visualized the calculated DAC of 22 output DEGs for each signal input and the aging-related DEG expression (Fig. 4B).

We also investigated candidate drugs that reversely regulate the aging-related expression of the DEGs found in this study, based on a LINCS database^34^. Of the 12,328 genes with available expression changes in the LINCS database, 19 of the 22 DEG markers found in this study were included. We investigated the drugs and conditions that can reversely regulate these 19 DEGs, and identified 11 candidate drugs, which induced the opposite direction of expression change for more than half of the DEGs (Fig. 4C). We visualized the drug-induced expression change of 19 DEGs and their aging-related expression changes observed in the RNA-seq analysis (Fig. 4D).

### Validation of candidate control targets

We investigated the binding targets of the candidate drugs identified with LINCS database to determine whether the drugs regulate the DEG expression by binding to the candidate targets using the PubChem database^35,36^. We obtained a list of assays in which drug-target binding, and agonist/antagonist experiments were conducted for the LINCS dataset candidate drugs. However, among the eight candidate target genes (Fig. 4A, E), high throughput screening assays were conducted only for NR3C2 and JUN, which can determine target-binding drugs and examine whether they have agonistic or antagonistic action. In the case of NR3C2, 243 assays were conducted for 506 drugs; however, the 11 candidate drugs identified from the LINCS database were not examined in PubChem assays. On the other hand, out of 69 assays where the binding ability of JUN to 7857 drugs was tested, curcumin, which has been found to notably modulate 10 aging-related DEGs in the LINCS database, was observed to bind to JUN and act as an antagonist. Previous studies have shown that administering curcumin increases the life span of model organisms such as mouse^37^, Drosophila^38,39^, and *Caenorhabditis elegans*^40^; moreover, numerous studies have been conducted on curcumin as a candidate target in aging^41,42^. For the other candidate genes, PubChem assays were conducted to examine siRNA activity instead of drug compounds, and we could not directly investigate whether drug-target binding occurred. To validate the candidate genes whose drug targets were not directly confirmed due to the lack of drug binding experimental data on the PubChem BioAssay, we further investigated the connection between them and aging via a literature survey; we found changes in aging-related phenotypes following changes in the expression level of relevant genes predicted by signal flow analysis (Table 1).

**Table 1:** Supporting evidence from the literature regarding candidate genes that regulate aging.

Candidate target	Change in expression	Aging-related phenotype	References
GMEB1	↓	Inhibition of cell growth in non-small cell lung cancer (human); increased expression upon nicotine exposure after smoking (human)	^70,71^
NR3C2	↓	Reduced risk of high blood pressure and skin aging (mouse); downregulated by Sirt-1 (monkey)	^72–74^
JUN	↓	Binding target of curcumin (mouse, human); increased life span through JUN downregulation (worm, fly, mouse)	^37–40,75^
KLF4	↓	Inhibition of cell senescence in bone marrow stem cells (human); improvement in heart function (human)	^76,77^
FOXJ3	↓	Facilitates neurogenesis (jellyfish); facilitates differentiation of embryonic stem cells into neurons (mouse)	^78,79^
NFYB	↑	Increased life span (worm); impaired regulation in Parkinson’s and Alzheimer’s diseases (human)	^80,81^
ZBTB33	↑	Maintenance of vascular endothelial cell homeostasis (human) and blood pressure homeostasis (mouse)	^82,83^
ZEB1	↑	Reduced expression upon UV ray-induced skin irritation (human); reduced risk of myoatrophy (mouse)	^84,85^

## Discussion

Recent advances in multi-omics integrative analysis have facilitated a detailed understanding of aging-related diseases and the development of therapeutic strategies. Numerous studies have used multi-omics data to estimate chronological age^43^, identify distinct stages of aging^44^, investigate interconnectivity of aging-related diseases^45^, and discover anti-aging drug compounds^46^. Many of these studies integrated multi-omics data using network approaches; however, there are limitations to analyzing undirected networks based on similarity, conditional dependency, or co-expression as exploring causal relationships among biomolecules is unfeasible. In the present study, we conducted methyl-seq and RNA-seq analysis as well as integrated multi-omics analysis to investigate the regulatory mechanism between methylation and expression according to a GRN.

In network analysis, constructing an accurate network structure is crucial, particularly for signal flow analysis, which mainly relies on the topology information of the network. In general, GRNs that are constructed from databases such as TRANSFAC and STRING can be regarded as canonical networks containing the entire set of gene regulations that can be partially activated or deactivated depending on the conditions. To investigate the GRN in terms of aging, we reconstructed a subnetwork—based on a previously constructed blood-specific GRN—to include genes whose methylation and expression levels changed with age. The observation that TF genes affected by DMP markers modulate downstream genes in a direction consistent with DEG markers demonstrated the existence of a regulatory relationship between the two omics layers.

**Figure 5:**
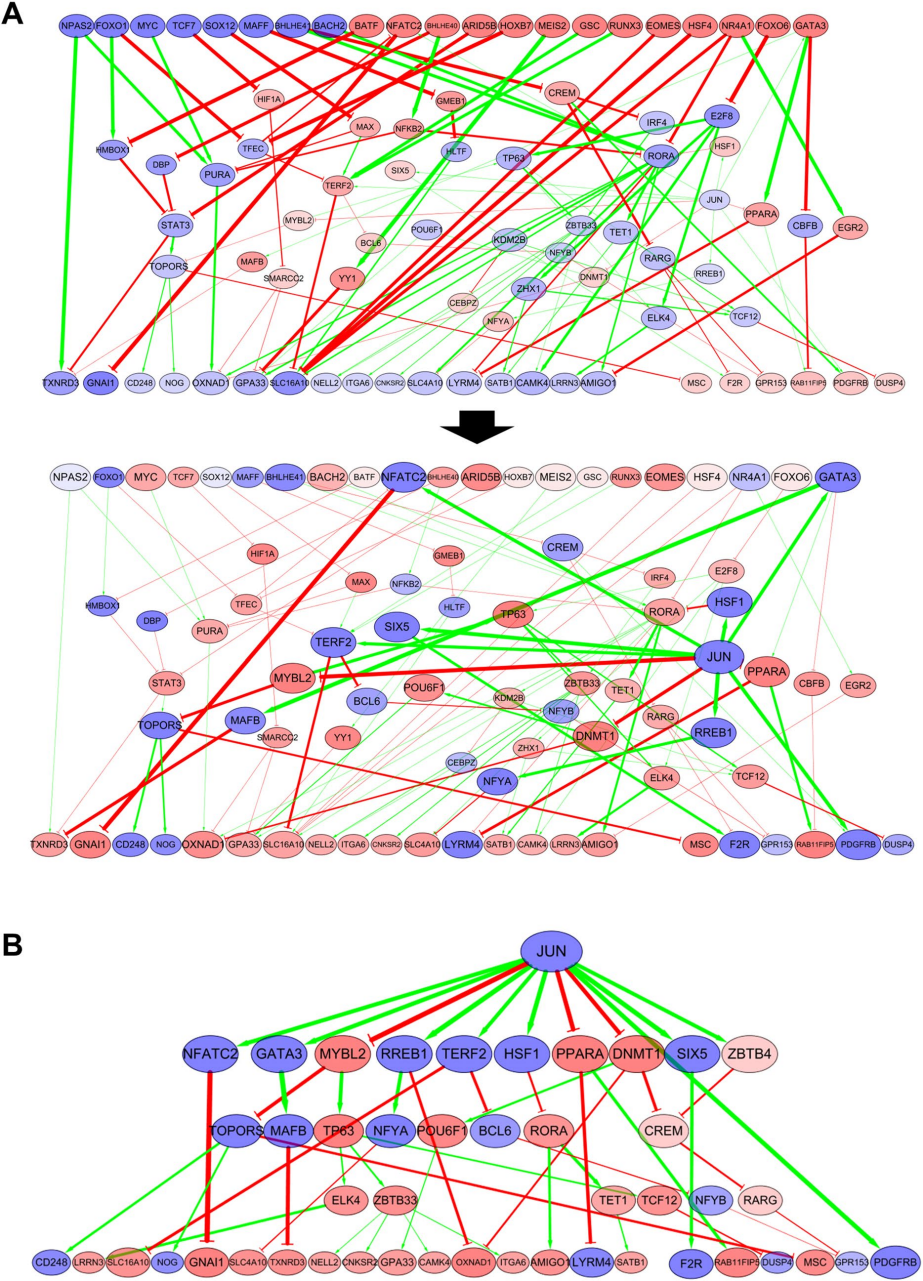
Subnetwork visualization. (**A**) Subnetwork showing the regulation of expression of DEG outputs by DMP inputs; 17 out of 22 DEG output nodes are reversely regulated by JUN inhibition. (**B**) A subnetwork showing the regulation of output DEGs by JUN inhibition and its downstream regulation. An increase in edge thickness and node size is based on the intensity of the signal transmitted to the DEG markers via the network. Up-regulated and down regulated genes are denoted with red and blue, respectively.

Based on this regulatory relationship, we identified the candidate control targets (i.e., intermediate nodes) that can reversely regulate the DEG markers. JUN, one candidate target identified via network analysis (Fig. 5A), was validated using the expression profile and binding assay data found in experimental databases. We then found that curcumin, which reversely regulated DEG markers, binds to JUN and inhibits its activity. This indicates that a connection to aging can be discovered by performing additional research or accumulating data on other candidate target genes that have not yet been sufficiently investigated.

In searching for direct downstream genes of JUN (Fig. 5B), we found that a few downstream genes are regulated in the direction of accelerating aging or promoting aging-related diseases (Table 2). However, whether controlling expression of a specific gene can regulate most downstream genes in the direction of reversing aging does not indicate that all downstream genes are regulated in a consistent direction. Several studies have reported that TF regulation of downstream genes can exert opposing effects on biological processes^47^ and that a TF can bi-directionally regulate a target gene depending on the cofactor to which it binds^48^; this is beneficial for achieving homeostasis. Therefore, candidate targets from computational studies should be examined in vitro and in vivo in animals and humans to identify their effects on phenotypes, side effects, and optimal doses in the target tissue.

**Table 2:** Aging-related phenotypes regulated downstream of JUN.

Downstream gene	Change in expression	Aging-related phenotype	References
NFATC2	↓	Reduced risk of inflammation in Alzheimer’s disease (mouse); maintenance of cardiac homeostasis (mouse)	^86,87^
GATA3	↓	Reduced expression by salidroside (fly, mouse, human); involved in B cell differentiation and immune response (mouse)	^88–91^
MYBL2	↑	Recovery of heart cell senescence after cardiac infarction (human); inhibition of cellular senescence in multiple cell types (human)	^92,93^
RREB1	↓	Inhibition of tumor suppression in multiple cancer types (human); a master regulator in Alzheimer’s disease (human)	^94,95^
TERF2	↓	Premature skin aging and increased risk of skin cancer (mouse); acceleration of telomere loss in fibroblasts (human)	^96,97^
HSF1**	↓	Proliferation of cells with damaged DNA (mouse); potential therapeutic target in cancer (human)	^98–100^
PPARA	↑	Increased risk of Alzheimer’s disease and cognitive decline (mouse); reduced risk of osteoarthritis (human)	^101,102^
DNMT1	↑	Recovery of UV-induced skin cell senescence (human); involved in ovarian aging and infertility (mouse)	^84,103^
SIX5	↓	Increased proliferation of muscle satellite cells (mouse); recovery of muscular dystrophic phenotype and life span (mouse)	^104,105^
ZBTB4*	↓	Facilitates tumorigenesis (mouse); inhibition of apoptosis in response to p53 activation (human)	^106,107^

*Downstream genes that appeared to be regulated in the direction of accelerating aging or promoting aging-related diseases.

Finally, regulation of gene expression in this network study only considered part of the epigenome and transcriptome; however, other omics layers involved in modulation before or after transcription, such as chromatin remodeling and protein conformation, can ultimately affect the behavior of biomolecules. Recently, multi-omics data across various other layers have been successfully accumulated on a large scale and with a longitudinal design^49,50^. When multi-omics network becomes complex and elaborate with the accumulation of data, other network theories can be applied such as reducing the size of biological networks^51^ or identifying robust control targets that are less affected by timing^52^. Additionally, clustering methods^53,54^ developed for time-series profiles can also be applied with the longitudinal data to infer regulatory mechanisms^55^ or functional annotations^56^ of molecular markers in each omics layer. Along with advances in theoretical frameworks, employing these qualitative and quantitative multi-omics datasets and phenotype data can improve integrated omics analysis of a network biology and provide new insights that could not be discovered at the level of individual markers or a single omics layer.

## Material and methods

### Study design and participants

Healthy adult volunteers between 20 and 80 years of age were recruited from two cities in Republic of Korea: Ulsan and Miryang by Welfare Genome Project (WGP)^57^. Of these, the 51 samples with 24 males (47.1%) and 27 females (52.9%) were sequenced. Their mean age was 44.73 ± 15.02 years. All participants took an empty stomach 9 h before blood collection. The blood samples were frozen at − 80 °C and 15 ml of them were used for Methyl-seq and RNA-seq. Smoking status of participants were collected using a questionnaire, and classified as “smokers” or “non-smokers” (including both former and current smokers). We adjusted for sex bias in identifying DMP and DEG markers, but not for smoking status, since only 8 of 51 participants were smokers and only males (Supplementary Fig. S4).

### Ethics, consent, and permissions

All 51 blood samples were obtained from Ulsan University Hospital, and written informed consent was obtained from all participants. This study was approved by the Institutional Review Board of Ulsan National Institute of Science and Technology (approval no. UNISTIRB-16-13-C). All experiments were performed in accordance with relevant guidelines and regulations.

### DNA methyl-sequencing

Genomic DNA was isolated from blood using the DNeasy Blood & Tissue Kit (Qiagen, Hilden, Germany) according to manufacturer’s instructions. Extracted DNA was quantified using the Quant-iT BR Assay Kit (Invitrogen, Carlsbad, CA). Genomic libraries were prepared using the SureSelectXT Methyl-Seq Target Enrichment System for Illumina Multiplexed Sequencing (Agilent Technologies, Santa Clara, CA). Briefly, genomic DNA (3 μg per sample) was randomly sheared using an ultrasonicator (Covaris Inc., Woburn, MA), after which DNA fragments were extracted. Samples were then subjected to end repair, methylated adapter ligation, hybridization to SureSelectXT Methyl-Seq Capture Library, streptavidin bead enrichment, bisulfite conversion, and PCR amplification. Sample genomic libraries were indexed and pooled for multiplexed sequencing on an Illumina HiSeq 2500 platform (Illumina, San Diego, CA) using 101 bp paired-end reads.

### Read mapping and methylation analysis

The sequenced methyl-seq reads were filtered using the IlluQCPRLL.pl script of NGSQCToolkit (v. 2.3.3)^58^, and then reads with Q20 > 70% were used. Using Bismark (v.0.14.5)^59^, the filtered reads were mapped to the human reference genome (hg38; ENSEMBL v.95) and duplicated reads were removed. Methylation values at each site were obtained using MethylExtract (v.1.9.1)^60^. We filtered out sex chromosome regions to reduce sex bias. The methylation values were normalized per sample using MethylKit package in R (v.3.5.0)^61^, and adjusted for sex bias using Combat algorithm from SVA package^62^ in R (v. 3.5.0). Pearson’s correlation coefficient was calculated to identify aging-related DMPs. Multiple test corrections were not applied in the methyl-seq analysis due to the small sample size compared to the large number of CpG positions. To explore the regulatory impact of DMPs on TF expression, we calculated Pearson’s correlation coefficient between DMPs and the expression levels of their neighboring genes of which transcription start site or gene body region are located within 5 kb.

### RNA sequencing

Total RNA was extracted using the PAXgene Blood RNA Kit (Qiagen) according to manufacturer’s instructions. RNA quality was assessed using the Bioanalyzer 4200 system to determine the RNA integrity number and rRNA ratio. We purified mRNA from total RNA using polyA selection followed by fragmentation. The fragmented mRNAs were synthesized into single-stranded and double-stranded cDNA using random hexamer priming. RNA sequencing libraries were constructed from double-stranded cDNA using an Illumina TruSeq Stranded mRNA Library Prep Kit. Libraries of the 51 samples were sequenced on the Illumina HiSeq 2500 platform using 101 bp paired-end reads.

### Read mapping and expression analysis

The sequenced RNA-seq reads were filtered using the same criteria for methyl-seq analysis with the IlluQCPRLL.pl script of NGSQCToolkit. The filtered reads were aligned to the human genome (hg38; ENSEMBL v.95) by STAR (v. 2.6.0a)^63^. Gene expression was calculated with the htseq-count tool of the HTSeq software suite (v. 0.11.2)^64^. DEGs, whose expression varied significantly with age (treated as a continuous variable), were identified with adjusting for sex bias by DESeq2 (v. 1.26.0)^65^. DESeq2 calculates the log2 fold change of gene expression per unit of age (year) using generalized linear models. Adjusted P value was calculated based on Benjamini–Hochberg correction^66^.

### Blood tissue-specific GRN

To investigate the regulatory relationship between the DMP and DEG markers identified in the blood samples, a blood tissue-specific GRN constructed with the Passing Attributes between Networks for Data Assimilation (PANDA) algorithm^67^ was used. PANDA is an algorithm that can infer the structure of a GRN from a certain gene expression profile based on previously known TF-target gene binding and protein–protein interaction information. Using 38 tissues in the Genotype-Tissue Expression (GTEx) database^68^, Sonawane et al.^32^ constructed tissue-specific GRNs, from which we downloaded the blood tissue-specific GRN (https://zenodo.org/record/838734#.XALkry3MxTZ) and reconstructed an aging-related subnetwork that comprised our identified DMP and DEG markers. Based on our RNA-seq experiment, we refined the subnetwork by filtering out lower 5% genes in their expression variance among 51 blood samples as well as regulations whose correlation and partial correlation of TF-target gene expression were not significant (P > 0.05), so that the refined network structure can better reflect the expression profiles conducted in context of aging.

### Determining the direction of gene regulation

In the aging-related GRN constructed based on the GTEx-PANDA network, edges include directional information in terms of source and target; however, they do not include information on the regulatory mode that informs whether a source node promotes or inhibits a target node, which is required to apply signal flow analysis. To explore the regulatory mechanism of gene expression, we assigned the mode of action of each edge according to the correlation of expression levels observed in the RNA-seq experiment of the gene pair to which each edge connects to from the established aging-related GRN. In the aging-related GRN, which has a dense structure of regulatory edges, one target gene was often regulated by multiple TFs. This complex regulatory structure renders the correlation between target gene expression and a certain TF less significant because changes in target gene expression levels via regulation by other TFs acts as noise. In case of a target gene regulated by multiple upstream TF genes, to identify the direction in which a certain TF regulates the target gene, we assigned the mode of action in the direction that corresponds to the partial correlation coefficient^69^, via which expression changes regulated by other TFs were excluded. Edges with no significant partial correlation were excluded from subsequent analysis.

### Signal flow analysis

Signal flow analysis^27^ is an algorithm that can predict the influence of input signals from specific nodes on the other nodes based on the network structure and the mode of action of each regulation. To use the methylation states of DMP markers as expression signal input given to TF genes in the GRN, the beta values measured in 51 blood samples were normalized as Z-score for each position, and converted into values in a range between − 1 and + 1. DACs of output DEGs were calculated by signal flow analysis. The DEGs whose DAC were concordant with the aging-related expression change in the RNA-seq experiment were considered to be potentially regulated by DMP markers. To determine candidate molecular targets that can reversely regulate the 22 significant DEGs, + 1 and − 1 input signals were assigned to each intermediate node except for the DMP input and DEG output nodes. The DEGs whose DAC were opposite to aging-related change were considered to be controlled in direction to reverse aging. Signal flow analysis was performed with a default parameter (α = 0.5), which adjust the influence of signal flow from upstream genes and the basal activity of their target gene. DACs of output DEGs were calculated as previously described^27^.

### Drug-target profile database

To determine candidate drugs that can reverse DEG marker expression, we used the LINCS database^34^, which provides the expression level changes of 12,328 genes for a given drug perturbation under different doses and time conditions in various cell lines; expression levels of 978 landmark genes were measured using an array and those of the other genes were inferred from the landmark genes. We downloaded the level 5 modZ score of blood cell-derived B266 cell lines from GEO(GSE92742) data, which included 271 conditions of 47 drug perturbations at different dose and time conditions. We transformed the modZ score of the expression level into percentiles, and counted the number DEGs regulated in the opposite direction to changes with aging with a greater influence than those of the upper/lower tertile (Fig. 3C,D). To compare the capacity for reverse regulation of aging-related DEGs among the different drug compounds, we selected the dosage and time condition that controls the most DEGs in the opposite direction of aging in each drug perturbation.

To validate the candidate targets identified by signal flow analysis, we used PubChem Bioassay^35,36^, which provide the results of quantitative high-throughput screening assays, wherein a fluorescent target protein reacts with different doses of drug perturbations, and the trend of changes in fluorescence and cell viabilities are measured to examine whether the protein binds to the drug and exhibits an agonistic or antagonistic function.

## Supplementary Information

Supplementary Figures.

Supplementary Tables.

## Data Availability

Raw sequencing data of methyl-seq and RNA-seq analysis are freely available upon request and after approval from the Korean Genomics Center’s review board of UNIST. Information regarding data sharing can be found at http://koreangenome.org.
