# Supplementary material for: Identifying molecular targets for reverse aging using integrated network analysis of transcriptomic and epigenomic changes during aging
Source: Sci Rep. 2021 Jun 10;11:12317. doi: 10.1038/s41598-021-91811-1 (PMC8192508; doi:10.1038/s41598-021-91811-1)
Supplement: Supplementary file 1 — Supplementary Figures. [file 41598_2021_91811_MOESM1_ESM.pdf]

Supplementary Figures

Identifying Molecular Targets for Reverse Aging Using Integrated Network Analysis of Transcriptomic and Epigenomic Changes during Aging

Hwang-Yeol Lee[1,2], Yeonsu Jeon[3,4], Yeon Kyung Kim[3,4], Jae Young Jang[3,4], Yun Sung Cho[2], Jong Bhak[2,3*], Kwang-Hyun Cho[1*],

[1] Department of Bio and Brain Engineering, Korea Advanced Institute of Science and Technology (KAIST), Daejeon 34141, Republic of Korea

[2] Genome Research Institute, Clinomics Inc, Ulsan, 44919, Republic of Korea

[3] Department of Biomedical Engineering, College of Information-Bio Convergence Engineering, Ulsan National Institute of Science and Technology (UNIST), Ulsan 44919, Republic of Korea

[4] Korea Genomics Center (KOGIC), Ulsan National Institute of Science and Technology (UNIST), Ulsan 44919, Republic of Korea[4]

[5] Personal Genomics Institute (PGI), Genome Research Foundation (GRF), Osong 28160, Republic of Korea

*Authors for correspondence: K.-H. Cho (ckh@kaist.ac.kr, lead contact), J. Bhak (jongbhak@genomics.org)

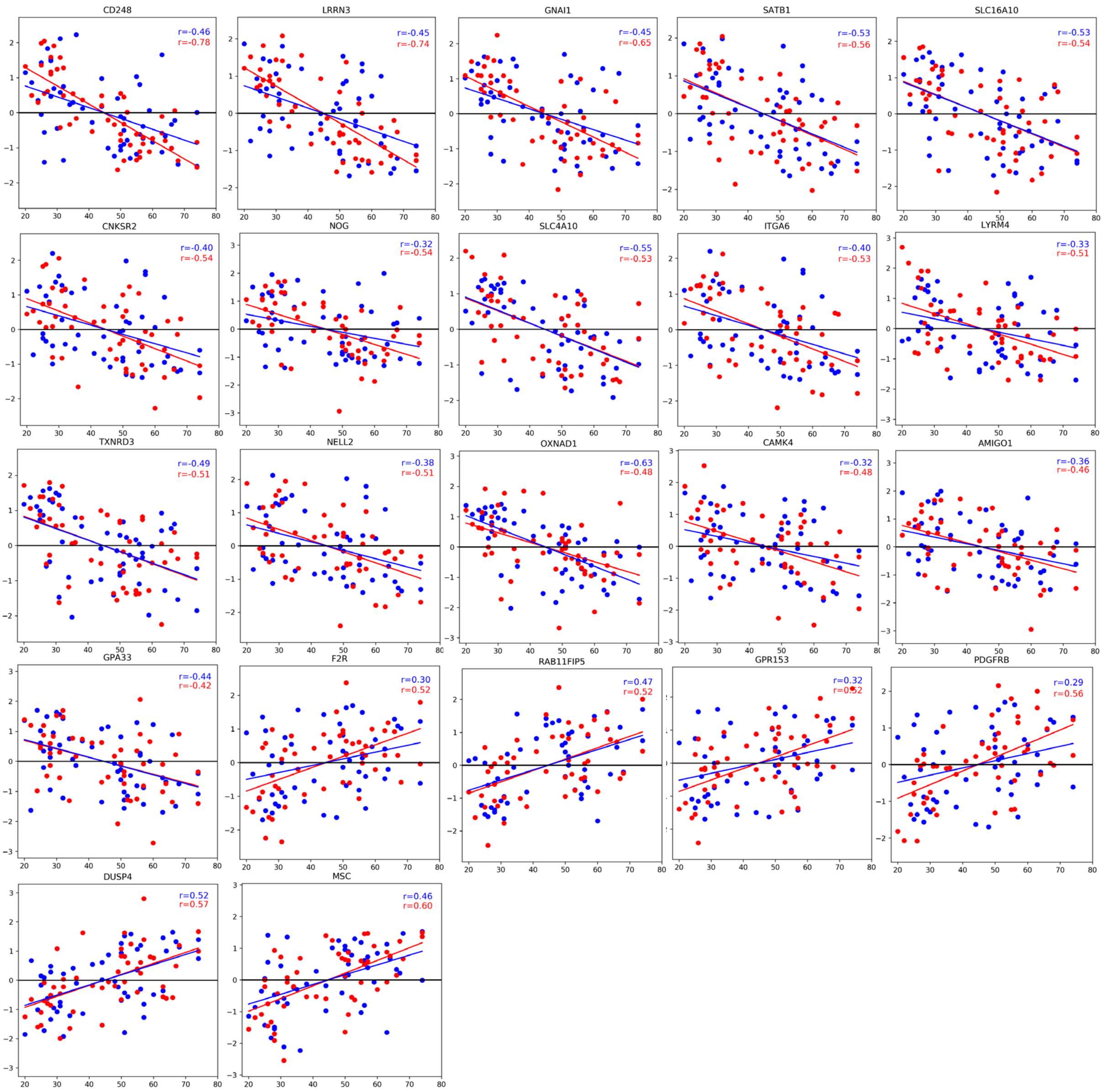

Supplementary Figure S1. **DACs from signal flow analysis and the aging-related fold changes in expression level from RNA-seq of 22 DEG markers, the change of directions for which were consistent.**

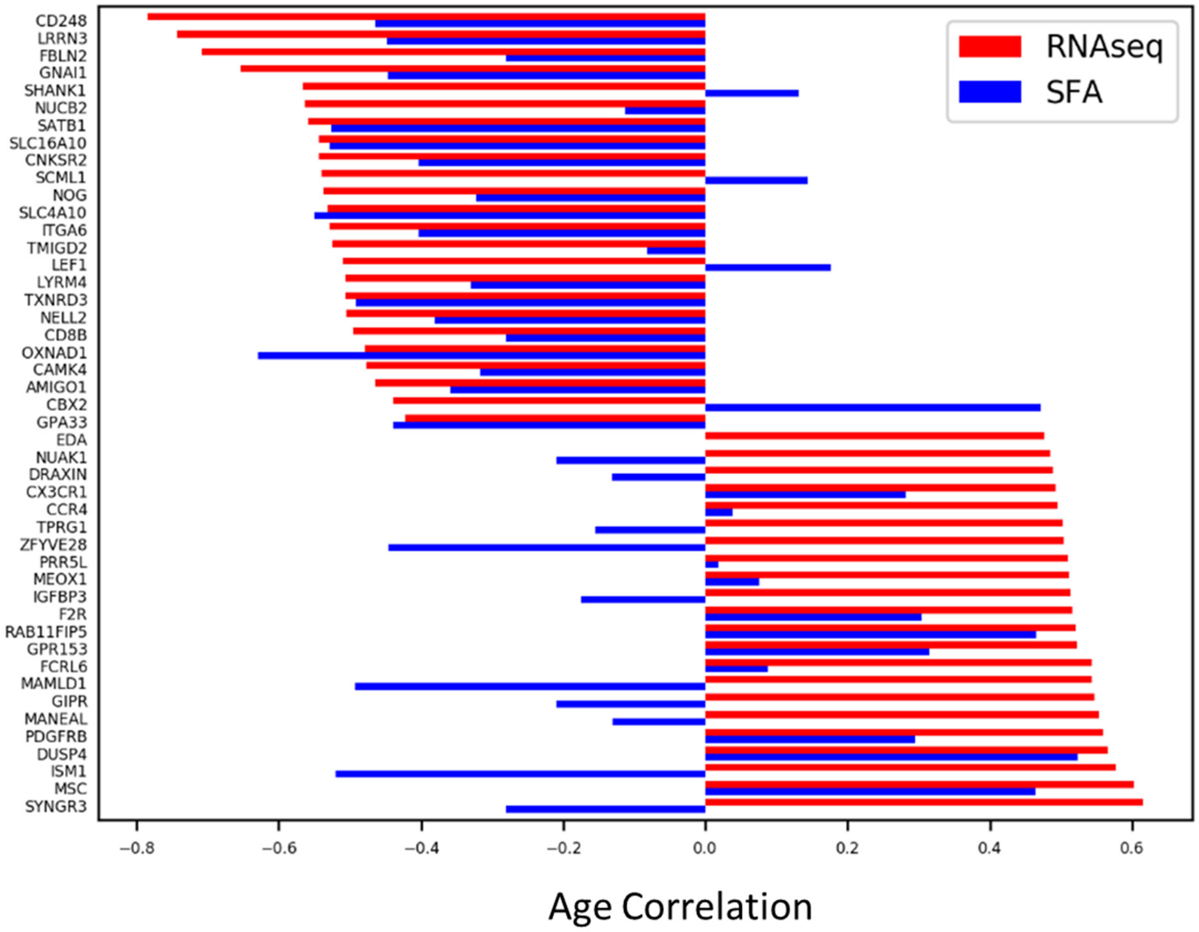

Supplementary Figure S2. **DACs and the aging-related fold changes of all DEG outputs including the ones for which the change of directions were insignificant or inconsistent.**

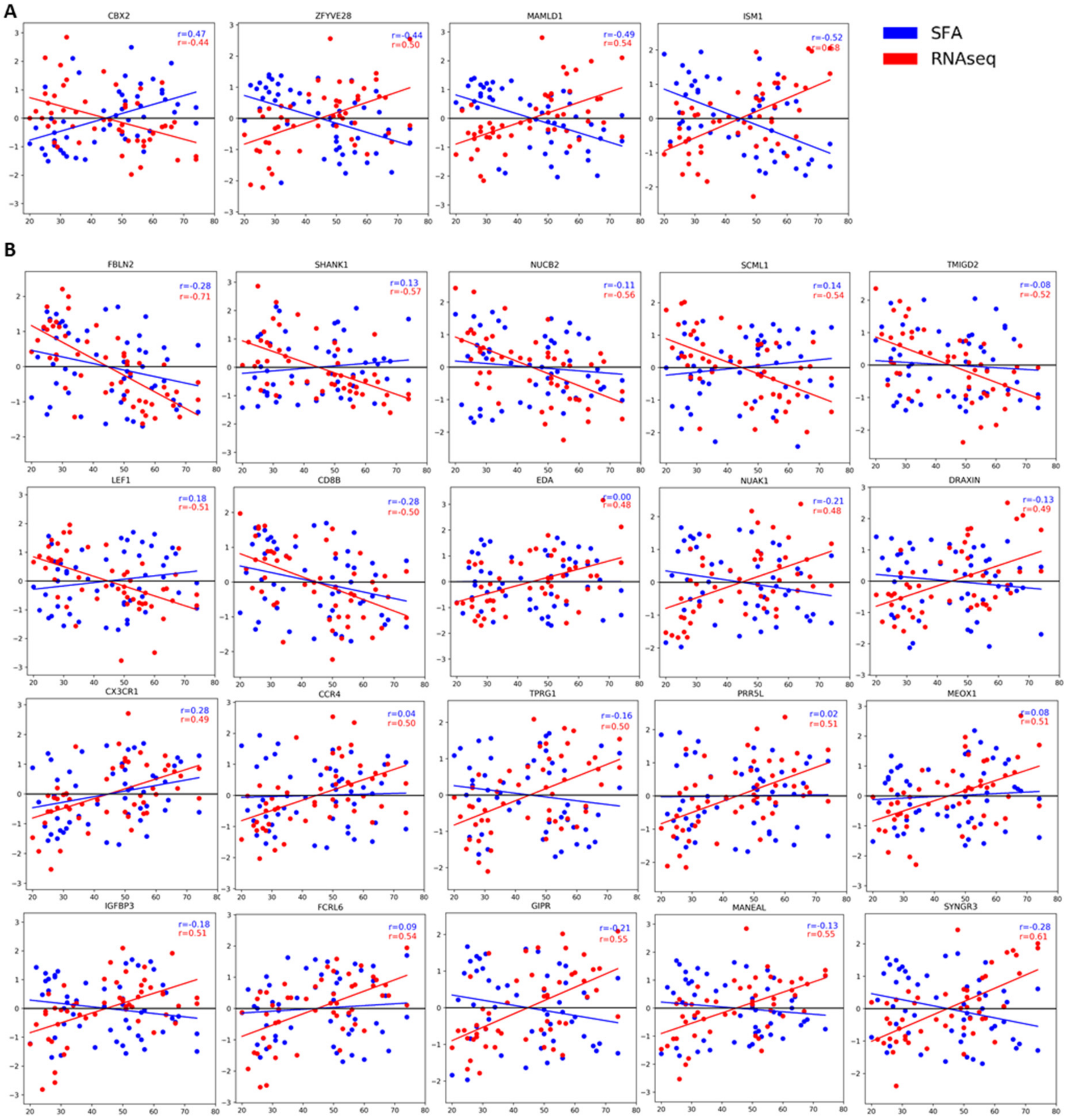

Supplementary Figure S3. **DACs from signal flow analysis and the aging-related fold changes in expression level from RNA-seq of 22 DEG markers for which the change of directions were (A) insignificant or (B) inconsistent.**

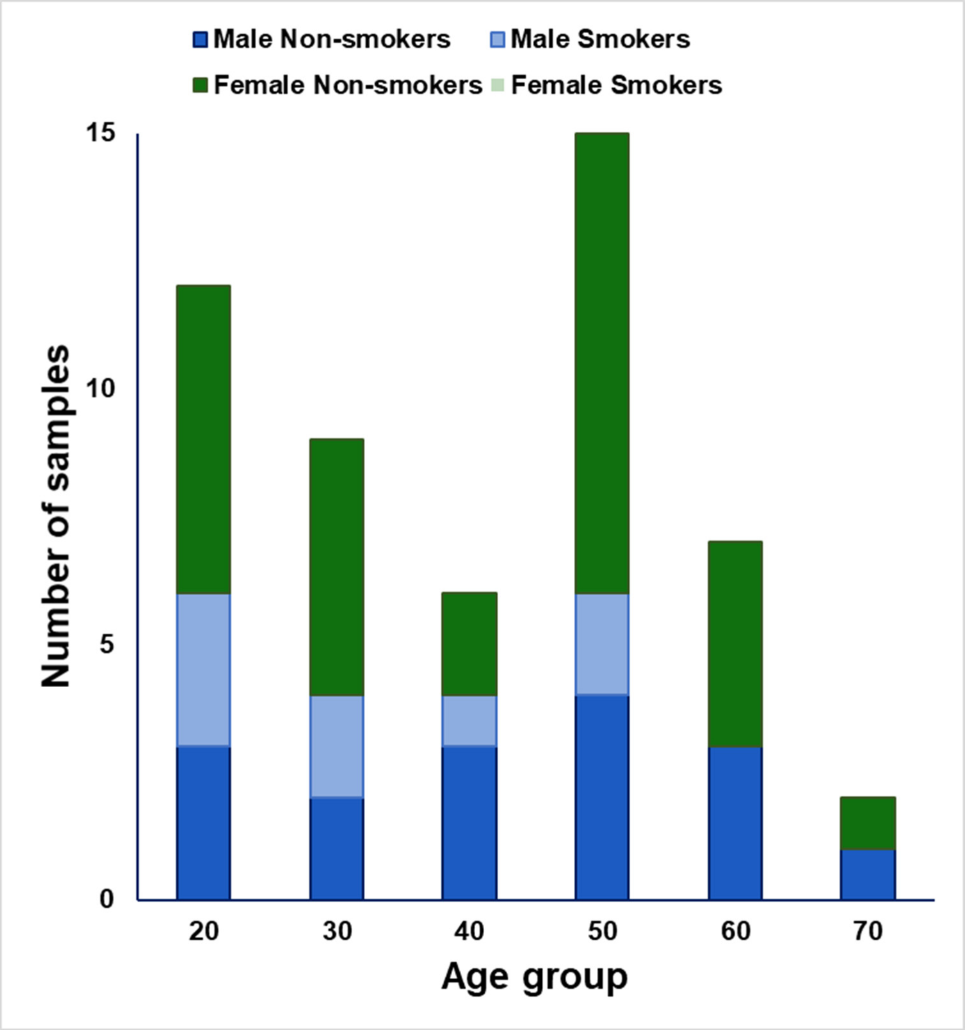

Supplementary Figure S4. **Distribution of age, sex, and smoking status in the study population.**
